# Supplementary material for: ProteinShader: illustrative rendering of macromolecules
Source: BMC Struct Biol. 2009 Mar 30;9:19. doi: 10.1186/1472-6807-9-19 (PMC2672931; doi:10.1186/1472-6807-9-19)
Supplement: Additional file 1 — ProteinShader program without source code. This compressed file contains the complete ProteinShader program including associated libraries, but no source code. A README.txt file gives an overview of the ProteinShader distribution, and the index.html file in the help subdirectory has directions on getting started with the program as well as a set of tutorials. [file 1472-6807-9-19-S1.zip › ProteinShader-beta-0_9_4-binary/help/api/org/proteinshader/gui/listeners/controlpanel/SelectorPanelListenerFactory.html]

SelectorPanelListenerFactory (ProteinShader API)


|  |  |  |
| --- | --- | --- |
| **Overview** \| **Package** \| **Class** \| **Use** \| **Tree** \| **Deprecated** \| **Index** \| **Help** | |  |
| **PREV CLASS**   **NEXT CLASS** | **FRAMES**    **NO FRAMES**     **All Classes** |  |
| SUMMARY: NESTED \| FIELD \| CONSTR \| METHOD | DETAIL: FIELD \| CONSTR \| METHOD |  |


---


## org.proteinshader.gui.listeners.controlpanel Class SelectorPanelListenerFactory

```
java.lang.Object
  org.proteinshader.gui.listeners.controlpanel.SelectorPanelListenerFactory
```

---

``` public class SelectorPanelListenerFactory extends Object ```

Creates the listeners for the components of the SelectorPanel.

---

**Constructor Summary**

`SelectorPanelListenerFactory(Mediator mediator,
SelectorPanel selector)`
  
          Constructs a SelectorPanelListenerFactory.


| **Method Summary** | |
| --- | --- |
| `WindowListener` | `createAtomDialogBoxListener()`             Creates a window listener for the Atom list dialog box. |
| `ListSelectionListener` | `createAtomListListener()`             Creates a list selection listener for the Atom list. |
| `ActionListener` | `createAtomsButtonListener()`             Creates an action listener for the "Atoms" button. |
| `ActionListener` | `createChainMenuListener()`             Creates an action listener for the Chain menu. |
| `ActionListener` | `createCloseButtonListener()`             Creates an action listener for the "Close" button on the Atom list dialog box. |
| `ActionListener` | `createModelMenuListener()`             Creates an action listener for the Model menu. |
| `ListSelectionListener` | `createResidueListListener()`             Creates a list selection listener for the Residue list. |
| `ActionListener` | `createResidueMenuListener()`             Creates an action listener for the Residue type menu. |

**Methods inherited from class java.lang.Object**

`clone, equals, finalize, getClass, hashCode, notify, notifyAll, toString, wait, wait, wait`

**Constructor Detail**

### SelectorPanelListenerFactory

```
public SelectorPanelListenerFactory(Mediator mediator,
                                    SelectorPanel selector)
```

:   Constructs a SelectorPanelListenerFactory.

    **Parameters:**: `mediator` - the centralized Mediator object that listeners call on to accomplish their task.: `selector` - the SelectorPanel that this class creates listeners for.


**Method Detail**

### createModelMenuListener

```
public ActionListener createModelMenuListener()
```

:   Creates an action listener for the Model menu. When the action
    listener is called, it will update the current Model on the
    Mediator and also update the Chain menu on the SelectorPanel.

    :   **Returns:**: The action listener.

---


### createChainMenuListener

```
public ActionListener createChainMenuListener()
```

:   Creates an action listener for the Chain menu. When the action
    listener is called, it will update the current Chain on the
    Mediator and also update the Residue list on the SelectorPanel.

    :   **Returns:**: The action listener.

---


### createResidueMenuListener

```
public ActionListener createResidueMenuListener()
```

:   Creates an action listener for the Residue type menu. When the
    action listener is called, it will update the Residue list on the
    SelectorPanel.

    :   **Returns:**: The action listener.

---


### createResidueListListener

```
public ListSelectionListener createResidueListListener()
```

:   Creates a list selection listener for the Residue list. When the
    listener is called, it will set the current Residue on the
    Mediator and call on the SelectorPanel to update the Atom list.

    :   **Returns:**: The list selection listener.

---


### createAtomListListener

```
public ListSelectionListener createAtomListListener()
```

:   Creates a list selection listener for the Atom list. When the
    listener is called, it will set the current Atom on the Mediator.

    :   **Returns:**: The list selection listener.

---


### createAtomsButtonListener

```
public ActionListener createAtomsButtonListener()
```

:   Creates an action listener for the "Atoms" button. When the
    listener is called, it will tell the Selector panel to open the
    Atom list dialog box.

    :   **Returns:**: The action listener.

---


### createCloseButtonListener

```
public ActionListener createCloseButtonListener()
```

:   Creates an action listener for the "Close" button on the Atom list
    dialog box. When the listener is called, it will hide the Atom
    list dialog box.

    :   **Returns:**: The action listener.

---


### createAtomDialogBoxListener

```
public WindowListener createAtomDialogBoxListener()
```

:   Creates a window listener for the Atom list dialog box. When the
    windowClosing() method of the window listener is called, it will
    hide the Atom list dialog box.

    :   **Returns:**: The window listener.


---


|  |  |  |
| --- | --- | --- |
| **Overview** \| **Package** \| **Class** \| **Use** \| **Tree** \| **Deprecated** \| **Index** \| **Help** | |  |
| **PREV CLASS**   **NEXT CLASS** | **FRAMES**    **NO FRAMES**     **All Classes** |  |
| SUMMARY: NESTED \| FIELD \| CONSTR \| METHOD | DETAIL: FIELD \| CONSTR \| METHOD |  |


---

# *Copyright © 2007-2008*
